# Early images of kuru and the people of Okapa

**DOI:** 10.1098/rstb.2008.4011

**Published:** 2008-11-27

**Authors:** D. Carleton Gajdusek

**Affiliations:** Institut Neurologique Alfred Fessard, Centre National de la Recherche ScientifiqueBâtiment 33, Avenue de la Terrasse, 91198 Gif-sur-Yvette, France

These photographs were mostly taken during the early days of my time in Okapa and our research on kuru, from March 1957. They are organized in sections, which show aspects of our research work, kuru patients, the care taken of patients by their families, kuru and other sorcery, and social aspects of the Fore people, including their housing, dress and ritual.

## 1. Research work

**Figure 1:**
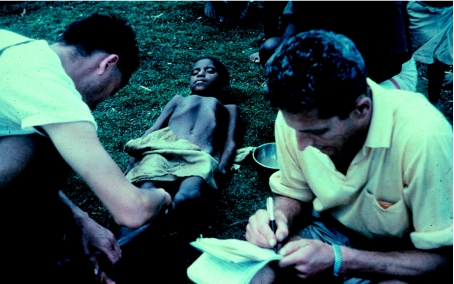
Dr Carleton Gajdusek (left) and Dr Vincent Zigas study a child patient with kuru at Okapa in 1957.

**Figure 2:**
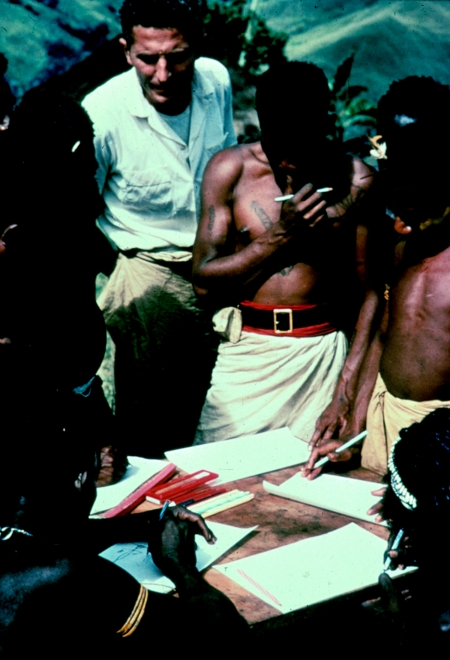
Dr Zigas (at back) watching children drawing for the first time in their lives, using coloured pencils and a flat planar surface to support the paper, brought by us. Agakamatasa village, South Fore, 1957.

**Figure 3:**
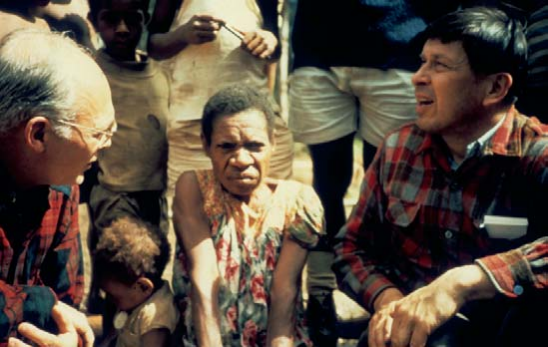
Dr C. Joseph Gibbs (left) and Dr Gajdusek with a kuru patient in Awande village, North Fore, in 1972.

**Figure 4:**
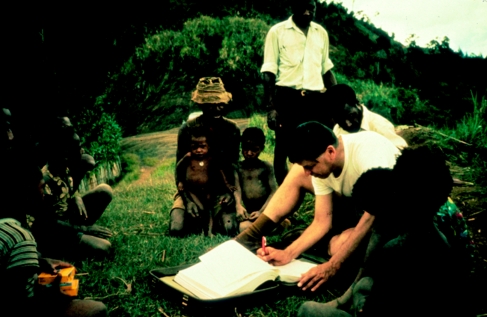
Dr Gajdusek revising the kuru record in the kuru database printout, Awande village, North Fore, in 1972.

**Figure 5:**
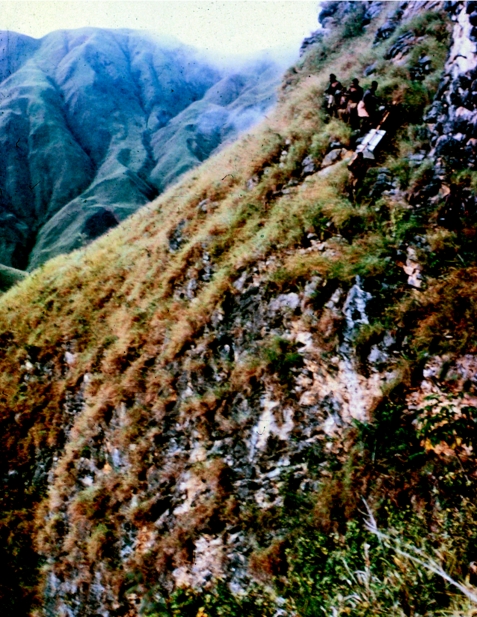
Patrol carrying metal ‘patrol boxes’ filled with patrol gear over difficult terrain, in 1960.

**Figure 6:**
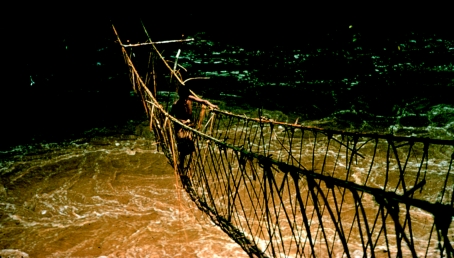
Patrol crossing a fast-flowing river by means of a bridge made of vines, in 1957.

figure 1figure 2figure 3figure 4figure 5figure 6

## 2. Kuru patients

figure 7figure 8figure 9figure 10figure 11figure 12figure 13figure 14figure 15figure 16figure 17figure 18figure 19

## 3. Care and attention given to patients

**Figure 20:**
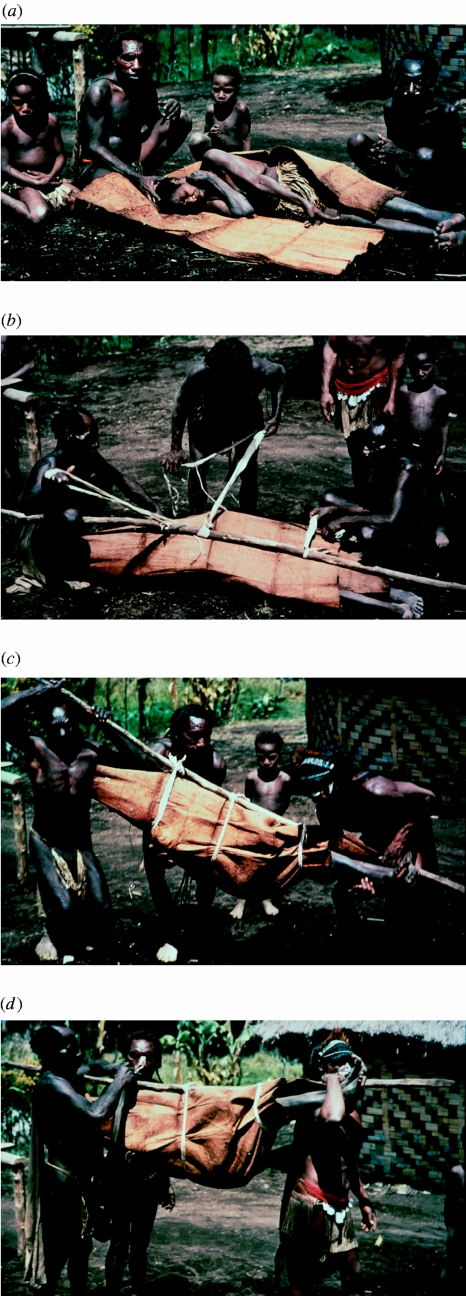
**–**) A kuru patient is wrapped in a beaten tapa cloth cape of mulberry tree bark, in which she will be carried to the hospital in Okapa, in 1957.

**Figure 21:**
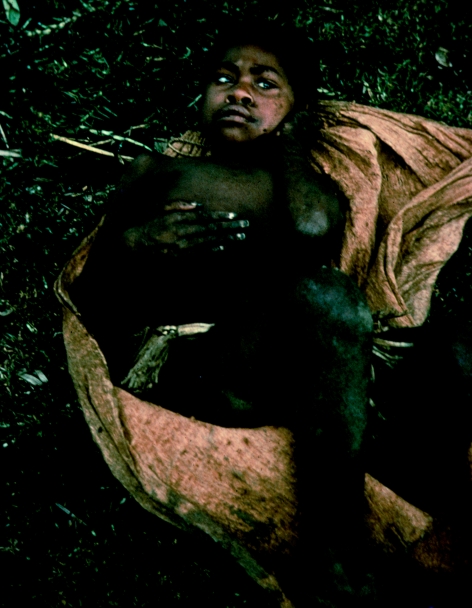
A boy with advanced kuru lying in a beaten bark cape in which he has been carried to seek medical attention, in 1957.

**Figure 22:**
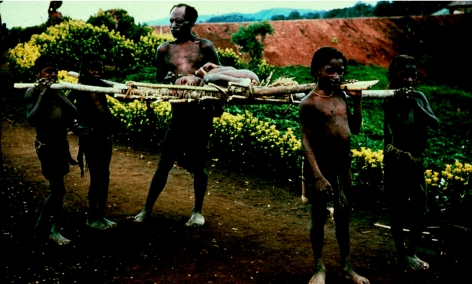
Four healthy small Fore boys carry their age-mate to the Okapa Kuru Hospital on a stretcher from distant Agakamatasa village in the South Fore, in 1957.

figure 20figure 21figure 22

## 4. Sorcery

figure 23

## 5. Aspects of Fore life and society

figure 24figure 25figure 26figure 27figure 28figure 29figure 30figure 31figure 32figure 33figure 34

## Figures and Tables

**Figure 7:**
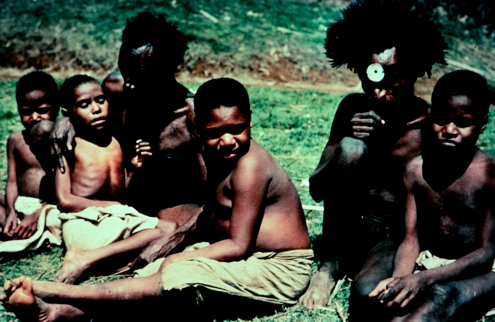
Four children with advanced kuru at Okapa, with two of their carers, 1957.

**Figure 8:**
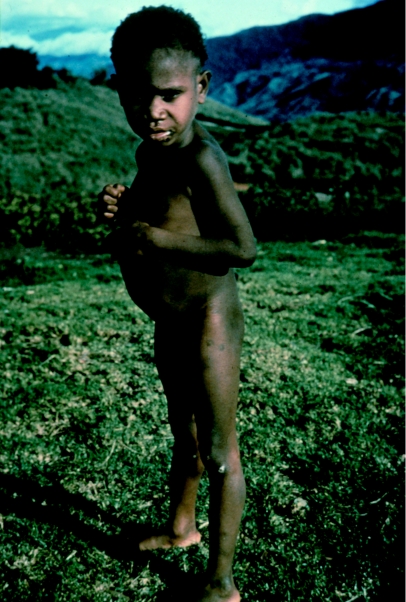
Yani, the youngest kuru patient at four-and-a-half years of age, in 1957.

**Figure 9:**
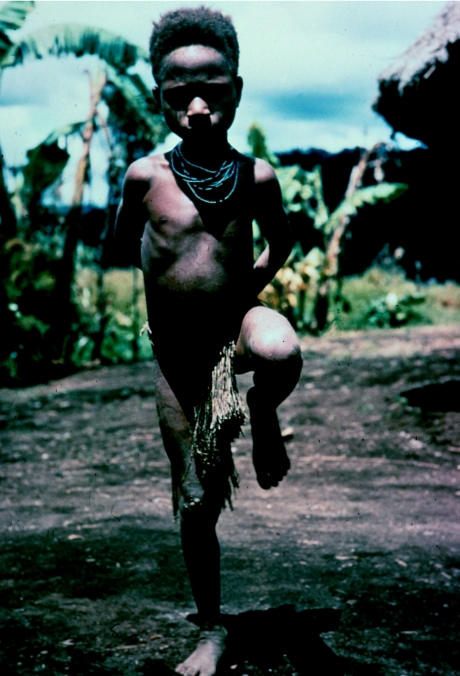
Amakiora, a girl with kuru, early in her course, in 1957.

**Figure 10:**
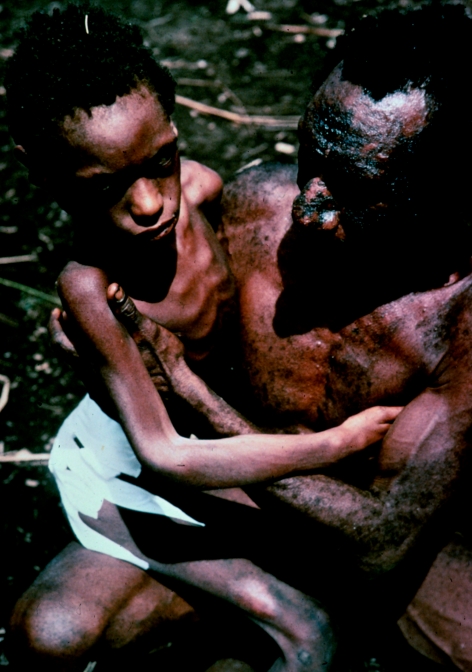
Amakiora, late in her course, in 1959.

**Figure 11:**
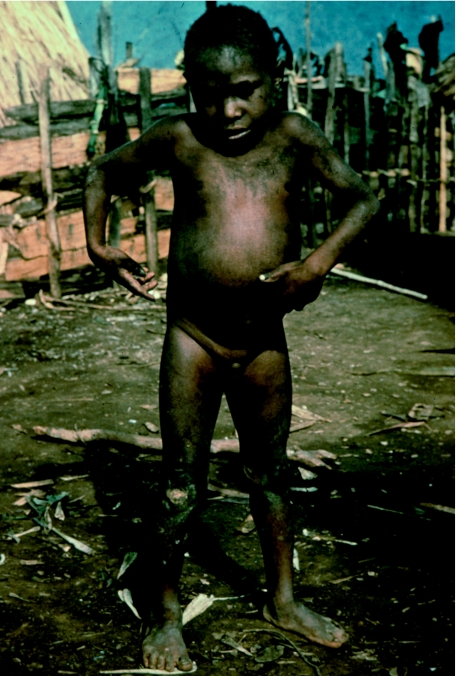
A boy with advanced kuru, caught in the midst of a myoclonic body jerk, in 1957.

**Figure 12:**
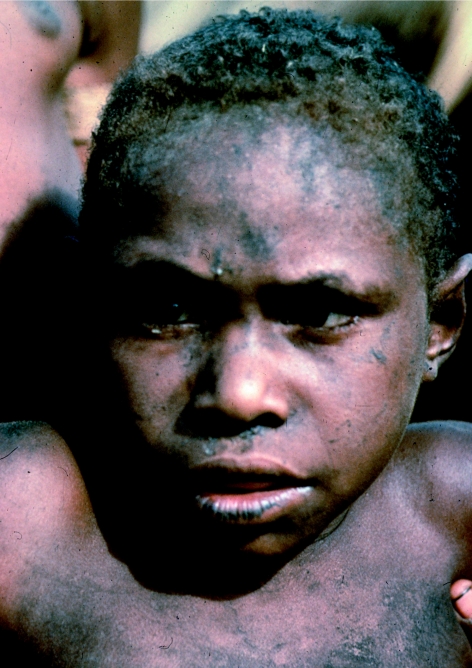
A boy with advanced kuru showing the shifting strabismus common in children with kuru, in 1957.

**Figure 13:**
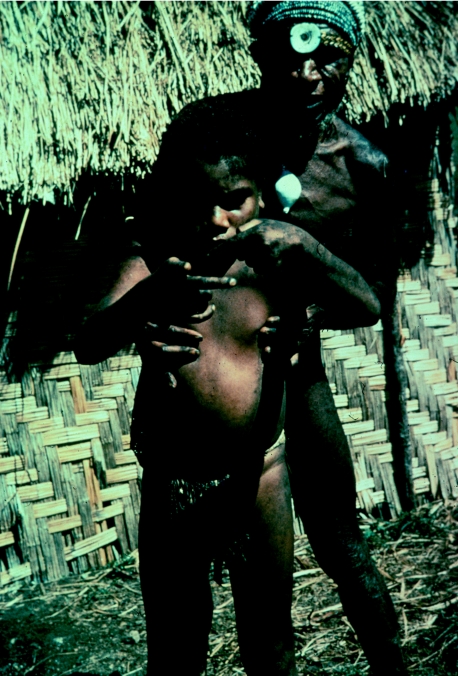
A boy from the Gimi linguistic group with advanced kuru, supported by his father, in 1957.

**Figure 14:**
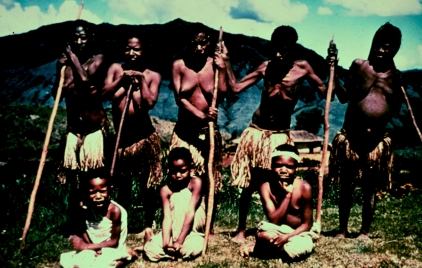
Five women with advanced kuru who require sticks for walking or standing and three girls (seated) with kuru, in 1957.

**Figure 15:**
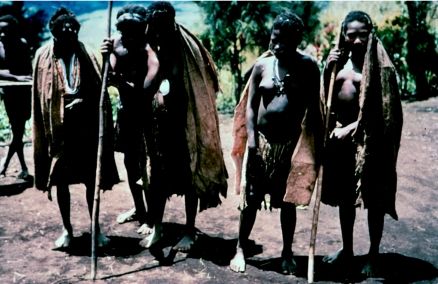
Women with kuru showing wide-based stance and astasia even when supported by a stick, in 1957.

**Figure 16:**
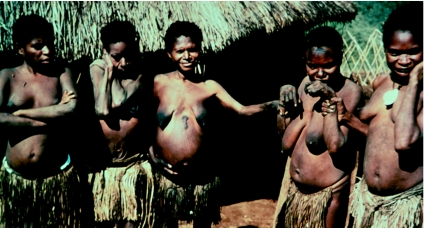
Five women with kuru showing upper limb postures adopted to prevent postural tremors, in 1957.

**Figure 17:**
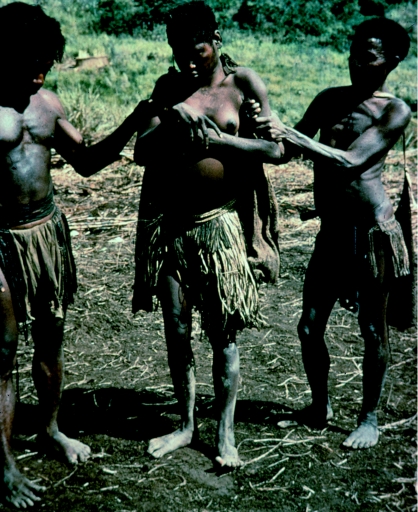
Five women with kuru showing upper limb postures adopted to prevent postural tremors, in 1957.

**Figure 18:**
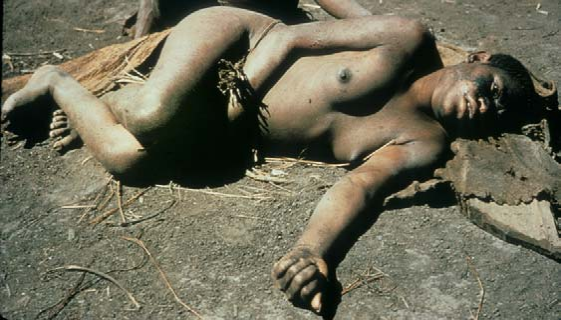
A woman with kuru in the terminal stage, unable to sit, being cared for outside her house, in 1957.

**Figure 19:**
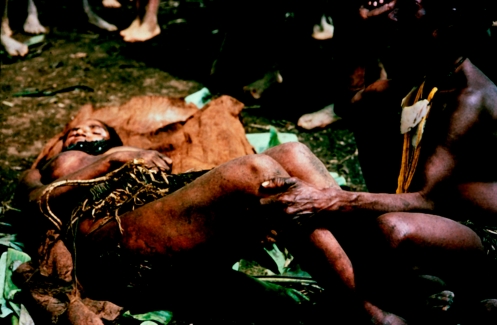
A woman who has just died of kuru showing deep decubitus ulceration, in 1957.

**Figure 23:**
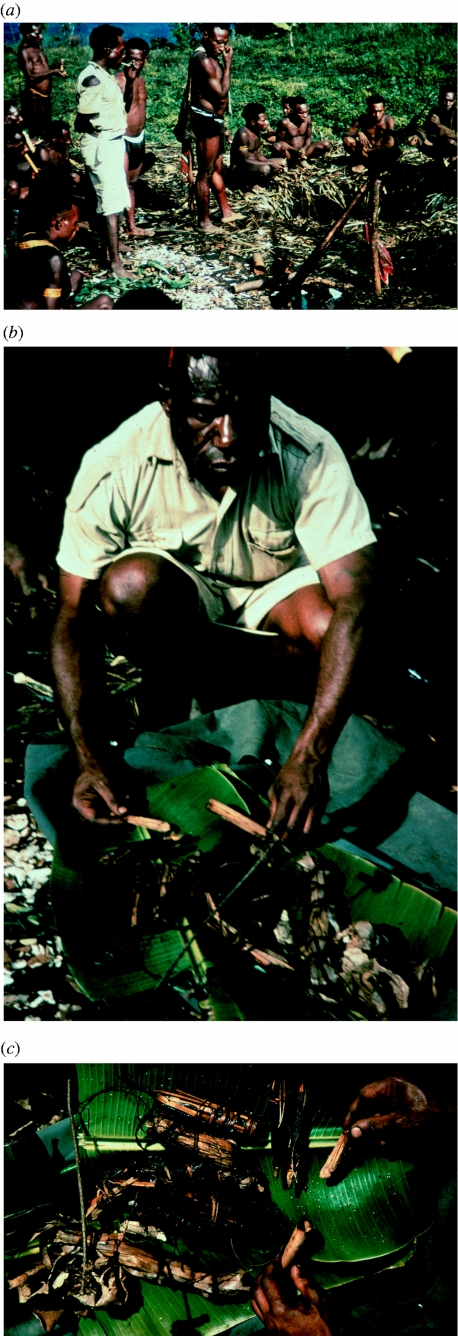
**–**) A large meeting held in early 1957 between Moke and Miarasa villages to disclose and destroy sorcerers' magic disease-producing packages for the three sorcery-induced diseases of kuru, tukabu and analisa.

**Figure 24:**
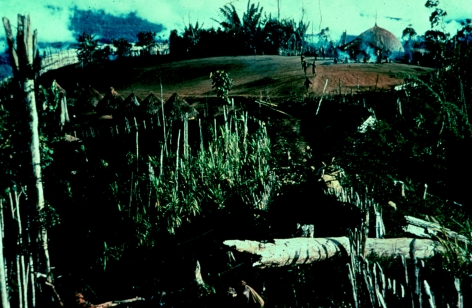
Waieti hamlet of Agakamatasa village, South Fore, in 1957. Row of *ambel anga* (women's houses) with menstrual huts behind them on the left. *Wae* (men's house) on the upper right. Fighting stockade in the background on the left.

**Figure 25:**
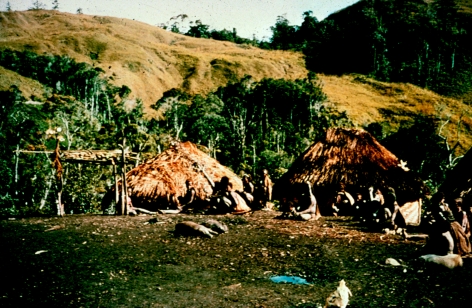
Fore women's houses (*ambel anga*), 1957.

**Figure 26:**
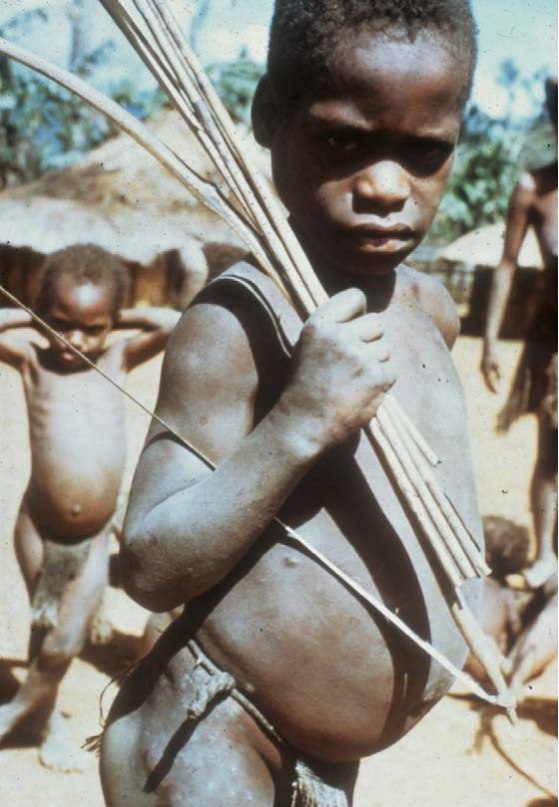
Fore *masi* (uninitiated boy) with his bow and arrows, 1960.

**Figure 27:**
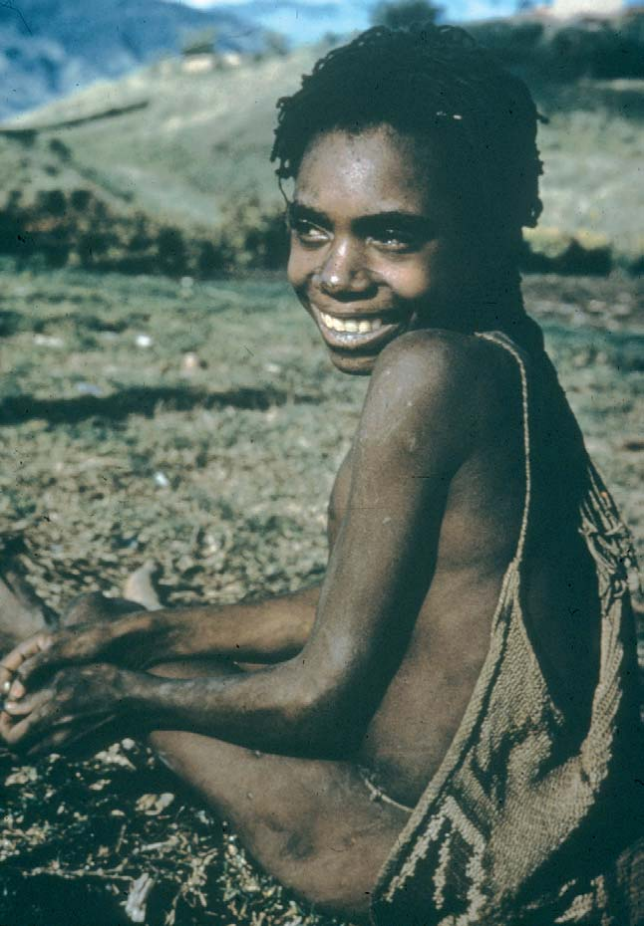
Wanevi Tubinaga at Pintogori, Okapa before his initiate's braided hair was cut, in 1957.

**Figure 28:**
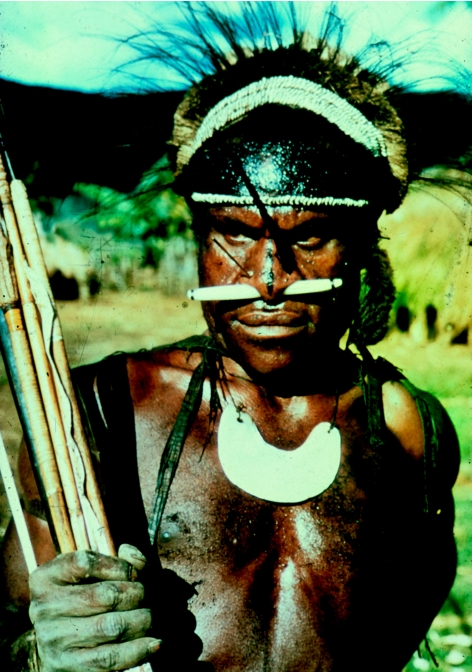
Fore warrior in 1957.

**Figure 29:**
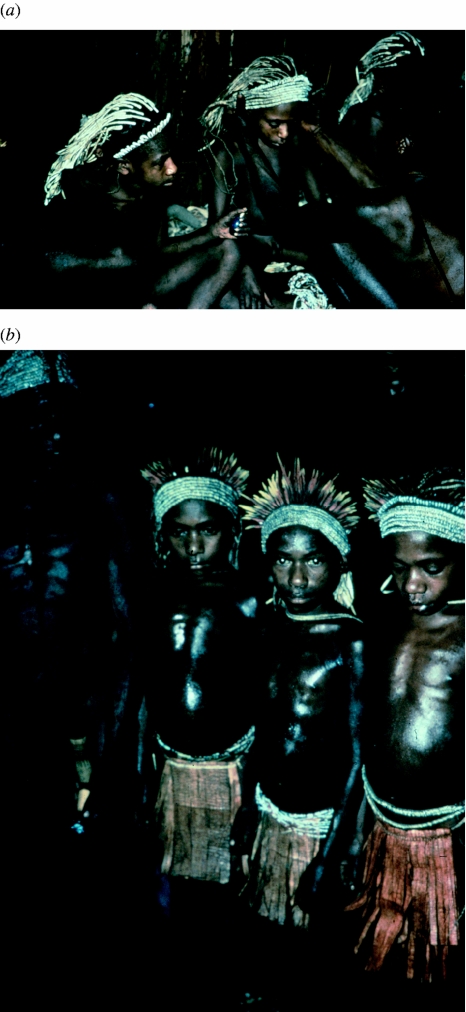
**) Three Fore boy initiates from *masi* to *mabi* in the first of three stages of Fore male initiations. They are anointed with pig grease, and new head ornaments of ‘tambu’ and ‘girigiri’ shells are fastened. Aga Yagusa village, North Fore, 1957.

**Figure 30:**
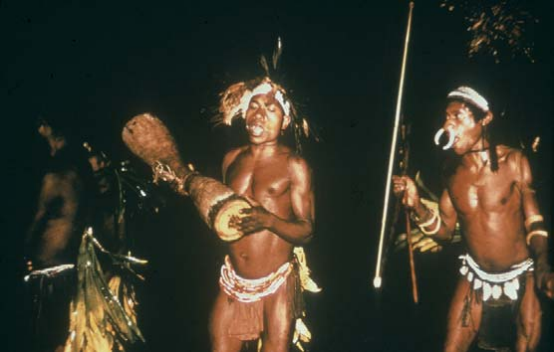
A dance and celebration in the evening, after the initiation, 1957.

**Figure 31:**
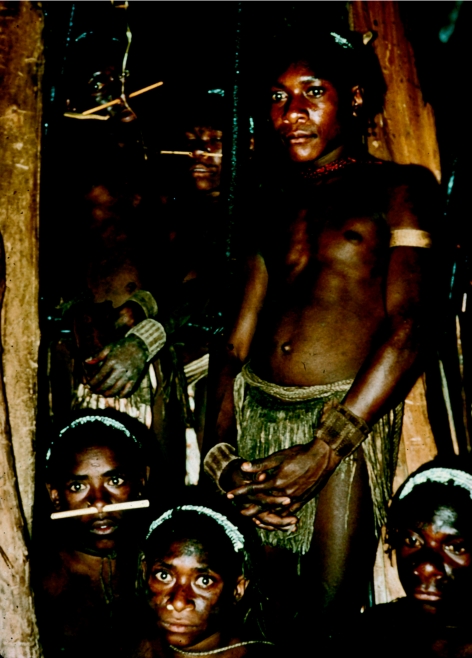
Six Fore *masimabi* (youths) in a *wae* (men's house), in 1957.

**Figure 32:**
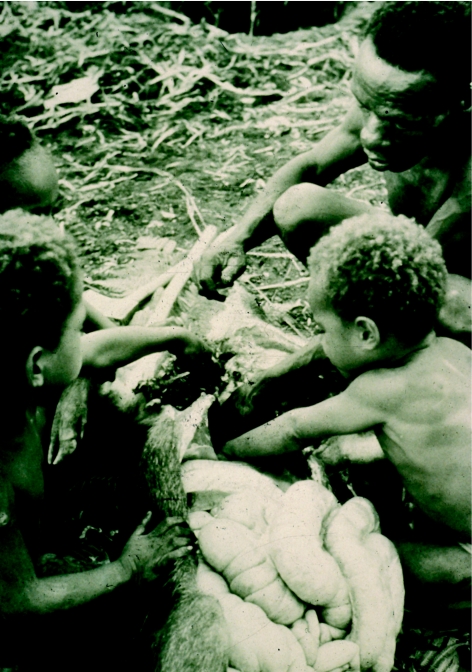
Small Fore boys collect blood from the open carcass of a pig killed for butchery a few minutes earlier, in 1957.

**Figure 33:**
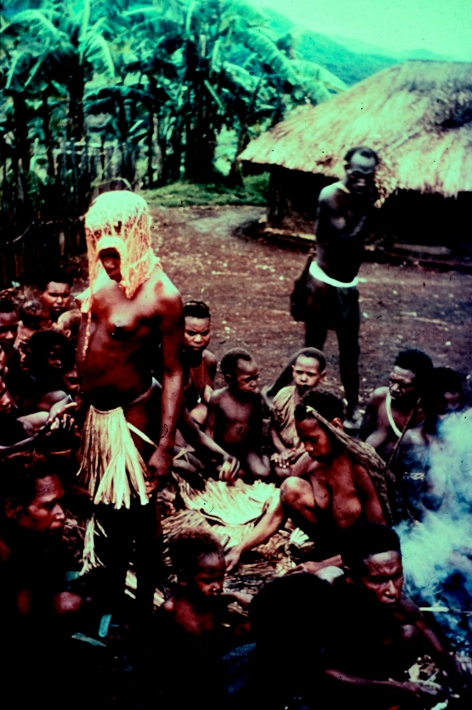
A Fore girl given in marriage, wearing a pig omentum draped over her head, in 1957.

**Figure 34:**
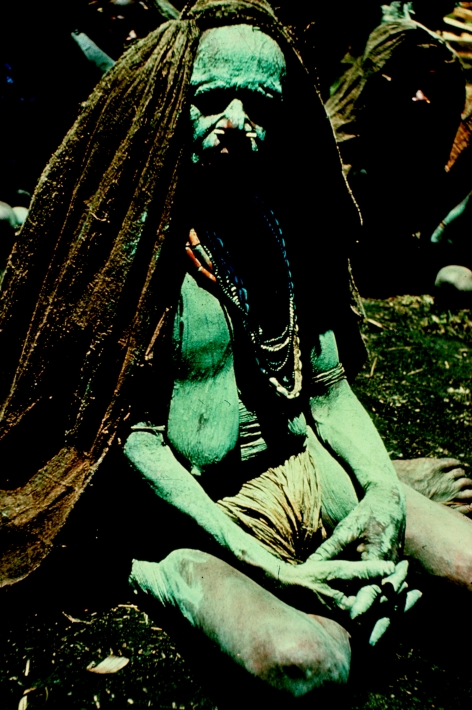
A woman in mourning covered with ashes, in 1957.

